# Functional State and Rehabilitation of Patients after Primary Brain Tumor Surgery for Malignant and Nonmalignant Tumors: A Prospective Observational Study

**DOI:** 10.3390/curroncol30050393

**Published:** 2023-05-22

**Authors:** Stanisław Krajewski, Jacek Furtak, Monika Zawadka-Kunikowska, Michał Kachelski, Jakub Soboń, Marek Harat

**Affiliations:** 1 Department of Physiotherapy, University of Bydgoszcz, Unii Lubelskiej 4, 85-059 Bydgoszcz, Poland; 2 Department of Neurosurgery, 10th Military Research Hospital and Polyclinic, 85-681 Bydgoszcz, Poland; 3 Department of Neurooncology and Radiosurgery, Franciszek Łukaszczyk Oncology Center, 85-796 Bydgoszcz, Poland; 4 Department of Human Physiology, Ludwik Rydygier Collegium Medicum in Bydgoszcz, Nicolaus Copernicus University, Karłowicza 24, 85-092 Bydgoszcz, Poland

**Keywords:** function, malignant, nonmalignant, primary brain tumor, postoperative complications, rehabilitation

## Abstract

The aim of this study was to compare the pre- and postoperative function of patients qualifying for resection of malignant and nonmalignant primary brain tumors to determine the relationship among tumor type, function, and the course of rehabilitation after surgery. This single-center, prospective, observational study recruited 92 patients requiring prolonged postoperative rehabilitation during their inpatient stay, who were divided into a nonmalignant tumor group (*n* = 66) and a malignant tumor group (*n* = 26). Functional status and gait efficiency were assessed using a battery of instruments. Motor skills, postoperative complications, and length of hospital stay (LoS) were recorded and compared between groups. The frequency and severity of postoperative complications, the time needed to attain individual motor skills, and the proportion of patients losing independent gait (~30%) were similar between groups. However, paralysis and paresis were more frequent in the malignant tumor group before surgery (*p* < 0.001). While nonmalignant tumor patients deteriorated more according to all scales after surgery, patients with malignant tumors were still characterized by worse ADL, independence, and performance at discharge. Worse functional outcomes in the malignant tumor group did not affect LoS or rehabilitation. Patients with malignant and nonmalignant tumors have similar rehabilitation needs, and patient expectation—especially those with nonmalignant tumors—should be appropriately managed.

## 1. Introduction

Central nervous system (CNS) tumors account for about 2% of all cancers [1,2,3]. Like with other cancers, the survival and functional state of patients with brain tumors depend on many factors, not least whether the tumor is benign or malignant, the histopathological subtype, and the genetic profile of the tumor [4,5]. Malignant brain tumors are more than twice as common as nonmalignant brain tumors [3]. While nonmalignant tumors do not usually infiltrate the brain tissue, they can still be symptomatic due to a mass effect [6].

The functional state and clinical condition of patients with brain tumors are often compared with patients after strokes [7,8,9,10,11,12,13,14,15] or craniocerebral injuries [14,16,17], and relatively few studies have explored functional outcomes according to tumor type. When reported, patients with malignant tumors usually score worse on relevant functional instruments than those with nonmalignant tumors [7,14,18,19]. Furthermore, surgery for malignant tumors is usually higher-risk, although patients with nonmalignant tumors at difficult locations may also experience significant decreases in performance [4,20,21].

Patients with a poor quality of life may not be suitable for surgery [22], and rehabilitation may be limited for these patients [19,23]. Indeed, rehabilitation is often a secondary consideration in this poor prognosis group due to disease severity, the need for adjuvant treatment, frequent recurrences, and potentially short survival. Most existing clinical practice guidelines for the management of brain tumors fail to include important aspects of rehabilitation [24,25]. However, rehabilitation can benefit even patients with poor function [26,27], and rehabilitation is important throughout the patient journey including in the early postoperative period [28], in long-term rehabilitation facilities [10,15], and at home [29,30,31].

There have been several attempts to develop rehabilitation programs for patients with poor quality of life [23,26,29], but these studies did not take tumor type into account. The impact of the operation itself has also only rarely been studied, i.e., the functional status immediately before the procedure and in the early postoperative period, which is often the starting point for rehabilitation. Determining the functional status throughout postoperative recovery would help us to understand the dynamics and effectiveness of rehabilitation, and taking tumor type into account may help to preselect patients for rehabilitation services and help to manage hospital resources. The assessment of functional status and independence at discharge is also important for planning future care and rehabilitation.

The primary goal of this study was, therefore, to compare the functional state, ADL, and motor skills (especially gait efficiency) of patients before and after primary brain tumor surgery, taking the malignant or nonmalignant nature of the tumor into account. We also sought to determine to what extent the tumor type determines the patient’s condition immediately after surgery. We further assessed the prevalence of complications affecting the rehabilitation course and time parameters such as the overall length of hospital stay (LoS), LoS after surgery, LoS in the intensive care unit (ICU), and the time of postoperative rehabilitation in the neurosurgery clinic.

## 2. Methods

### 2.1. Patient Cohort

**Figure 1:**
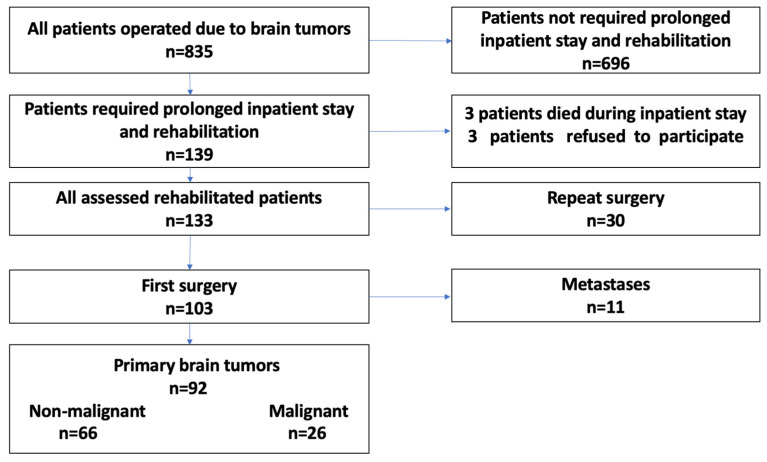
Flowchart of the study.

The Bioethics Committee at the Military Medical Chamber approved the study protocol (no. 164/18). The impact of tumor location and initial or repeat surgery on the course and results of rehabilitation in this cohort is discussed elsewhere [32,33]. Briefly, this was a single-center, prospective, observational controlled study (two intervention groups) with follow-up time from the day of admission to the clinic to the day of discharge. A total of 835 patients underwent operations for brain tumors between August 2018 and February 2020 (18 months). Patients with primary nonmalignant and malignant brain tumors operated on for the first time were evaluated. The tumor type was confirmed by histopathological examination (Figure 1).

The method of operation and access to the tumor were selected individually and optimally for each case on the basis of preoperative and intraoperative assessment by the operator. Head MRI with tractography and functional MRI were used to selected cases. The following methods were used intraoperatively: neuromonitoring in 56 operations (malignant tumors—18 (69.2%), nonmalignant tumors—38 (57.6%)), evoked potential monitoring in 31 cases (12 (46.2%) and 19 (28.85), respectively), 5-aminolevulinic acid (5-ALA) in six cases (6 (23.1%) and 0), and awake craniotomy in three cases (2 (7.7%); 1 (1.5%)). Three patients with malignant tumors were subjected to three of the above methods, nine had two, 11 had one, and three had none. Eleven patients with nonmalignant tumors used two of the above methods, 36 had one, and 19 had none.

The inclusion criteria for the nonmalignant tumor group (*n* = 66) were as follows: patients who underwent first brain tumor surgery; nonmalignant neoplasm; neurological deficits present; functional state worsened by surgery; need for prolonged rehabilitation. The inclusion criteria for the malignant tumor group (*n* = 26) were as follows: patients who underwent first brain tumor surgery; malignant neoplasm; neurological deficits present; functional state worsened by surgery; and need for prolonged rehabilitation.

### 2.2. Patient Assessment

The primary assessments were of functional status and motor skills, as described in our previous studies [32,33]. Each scale used assessed a different aspect of the functional state. Activities of daily living (ADLs) were assessed with the Barthel index (BI). This scale assesses self-reliance in eating, self-transferring, maintaining personal hygiene, using the toilet, washing, moving on flat surfaces and stairs, dressing, and controlling urine and bowel motions. General condition and performance living with cancer were assessed with the Karnofsky performance status scale (KPS). This scale assesses the impact of cancer on general wellbeing, professional functioning opportunities, and healthcare needs. The degree of self-reliance was assessed with the modified Rankin scale (MRS), which assesses the degree of disability and dependence on other people. Gait efficiency was assessed with the 10-point Gait index (GI), an easy-to-use scale used to assess gait disorders in everyday practice [32]. In the GI scale, 10 denotes a correct independent gait and 1 denotes impossibility to achieve upright vertical position; a score of 1–4 means that the patient does not walk, a score of 5–6 means that the patient walks with the assistance of another person, a score of 7–8 means that the patient walks independently with orthopedic equipment, and a score of 9–10 means that the patient walks on their own [32]. BI, KPS, and MRS are simple and commonly used instruments; their validity and reliability are well documented, and they have the sensitivity to detect clinically significant changes over time [34,35,36,37,38,39].

Patients were assessed using all four scales prior to surgery, immediately after surgery (day 2–3), and upon discharge. Time to discharge was 2–90 days. Individual motor skills assessed were passive sitting (the patient can spend time in a wheelchair), active sitting (independent sitting and stable trunk), independent standing, and independent gait with or without orthopedic equipment. Individual motor skills were noted before the operation and on which day after surgery the patient was able to perform each skill.

Secondary variables included the overall LoS, LoS after surgery, LoS in the ICU, the number of rehabilitation days, and the incidence of complications affecting the rehabilitation course. The Landriel Ibañez four-grade classification was used to assess the severity and type of complications affecting the course of rehabilitation. This classification has been used in several previous large studies on the topic, and it emphasizes its usefulness in assessing the impact of complications on length of hospital stay and quality of life. Grade I refers to non-life-threatening complications treated without invasive procedures; Grade II refers to complications requiring invasive management; Grade III refers to complications requiring treatment in the ICU; Grade IV refers to death due to complications. We distinguished complications directly related to surgical procedures and medical complications; however, due to their duration, complications were defined as temporary (up to 30 days after the surgical procedure) and permanent (lasting longer than 30 days) [40,41,42,43].

### 2.3. Statistical Analysis

All data are presented as the mean ± SD or number (percentage). The normality of the distribution of study variables was verified using the Shapiro–Wilk test. Relationships between categorical variables were determined with the chi-squared test. To investigate group and time effects on functional activity, we applied two-way repeated-measures analysis of variance (ANOVA) between groups (malignant tumor vs. nonmalignant tumor) and according to time (before surgery/after surgery/at discharge). Bonferroni’s test was used in the case of significant differences. A *p*-value < 0.05 was considered statistically significant. All analyses were carried out using Statistica 13.0 PL statistical package (StatSoft, Kraków, Poland).

## 3. Results

Of 835 patients with brain tumors, 139 (16.6%) required postoperative inpatient rehabilitation; three died during their inpatient stay, and three refused to participate in the study. Thirty patients underwent reoperation, and, among the 103 patients undergoing first surgery, 11 received operations for metastases. Finally, 92 patients with primary brain tumors who underwent first tumor resections were evaluated (Figure 1).

The majority (71.7%) of operations were for nonmalignant tumors: 48 were benign and 18 were low-grade (WHO grade II). The most common operation was for meningioma (22.8% of cases and 43.8% of benign tumors). Of the 28.3% of patients with malignant tumors, WHO grade IV glioblastoma was the most common, accounting for 61.5% of primary malignant tumors (Table 1).

There were no significant differences in gender, age, overall LoS, LoS after surgery, number of days in ICU, or number of rehabilitation days between the malignant and nonmalignant groups (Table 2). Every fourth patient had a postoperative complication. There was no difference between groups in severity of complications according to the Landriel Ibañez classification. In both groups, surgical complications were most common complications: bleeding into the ventricular system (*n* = 4), hydrocephalus (*n* = 6), postoperative hematoma (*n* = 3), and cerebrospinal fluid leakage (*n* = 4). Medical complications included cardiorespiratory failure (*n* = 2), dysphagia requiring PEG placement (*n* = 2), and pulmonary embolism and urinary tract infection (*n* = 1 each).

Temporary complications were more than twice as common as permanent ones in the nonmalignant tumor group, while, in the malignant group, this difference was smaller. Paralysis and paresis were more common in the malignant tumor group both before surgery (*p* < 0.001) and at discharge (*p* = 0.014; Table 3).

**Figure 2:**
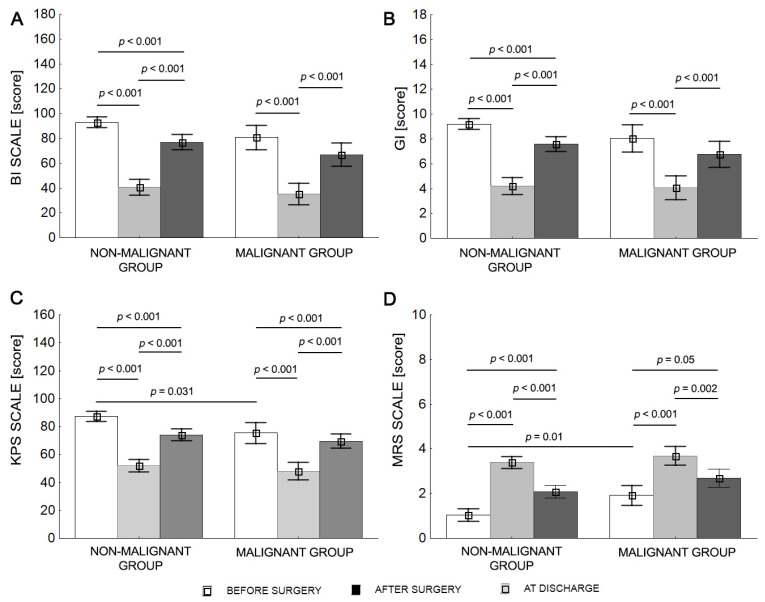
Mean values (±SD) for malignant and nonmalignant tumor patients before surgery, after surgery, and at discharge for BI (**A**), KPS (**B**), MRS (**C**), and GI (**D**).

Two-way repeated ANOVA revealed differences in groups at different timepoints (before surgery, after surgery, and at discharge) for the BI, KPS, and MRS scales. For the GI, there was a significant difference over time but not according to group. Before surgery, KPS values were significantly higher (*p* = 0.031) and MRS values were significantly lower in the nonmalignant tumor group than the malignant tumor group. After surgery, both the nonmalignant tumor group and the. malignant tumor group showed significantly lower BI, KPS, and GI scores and higher MRS scores compared with before surgery and at discharge. Similarly, at discharge, both groups were characterized by higher BI, KPS, and GI values, and lower MRS values compared with after surgery (*p* < 0.001). The BI and GI scores for the malignant tumor group were comparable before surgery and at discharge, *p* > 0.05 (Table 4 and Figure 2).

The mean BI score classified both groups as independent before surgery (BI > 80). After surgery, the nonmalignant tumor group patients required help (BI < 80), and the malignant tumor group patients required significant help (BI < 40). Both groups of patients required help to various degrees at discharge (BI < 80; Table 4).

The mean KPS score classified patients in both groups as able to carry on normal activities and work (KPS > 70) before surgery. The KPS score decreased after surgery, and patients in both groups were classified as unable to work, able to live at home, or care for most personal needs, with varying degrees of assistance (KPS 40–70). The KPS values were in the range of 70 for both groups at discharge (Table 4).

The mean MRS score classified the nonmalignant tumor patients as not significantly disabled and the malignant tumor group as slightly disabled prior to surgery. Nonmalignant tumor and malignant tumor patients had mean MRS scores of 3.4 and 3.7, respectively, after surgery, equating to moderate severe disability. At discharge, the mean MRS score was 2.1 for the nonmalignant tumor group (slight disability) and 2.7 for the malignant tumor group (moderate disability) (Table 4).

The mean GI score classified nonmalignant tumor patients as walking independently (GI = 9.2) and malignant tumor patients as walking with orthopedic equipment (GI = 8.0) before surgery. Neither group of patients could walk (GI < 5) after surgery, but nonmalignant tumor group patients could walk with orthopedic equipment at discharge, whereas malignant tumor group patients could walk with the assistance of another person for a several dozen meters at discharge (Table 4).

Comparing motor skills over the three periods, the largest difference between groups was found before surgery, especially for independent gait (*p* = 0.024). Motor skills decreased after surgery and were similar between groups, i.e., motor skills worsened more for patients with nonmalignant tumors. At discharge, all motor skills had improved compared with the postoperative period. Passive and active sitting and standing were achievable for a similar number of patients in both groups as before surgery (*p* > 0.05), but the possibility of independent gait decreased from 80.8% before surgery to 50.0% at discharge in the malignant tumor group (*p* = 0.013) and from 95.5% to 66.7% in the nonmalignant tumor group (*p* < 0.001; Table 5).

Four patients (15.4%) in the malignant tumor group and two (3.0%) in the nonmalignant tumor group could not walk even with the help of another person before surgery. After surgery, 14 (53.8%) in the malignant tumor group and 32 (48.5%) in the nonmalignant tumor group could not walk with help, and seven (26.9%) in the malignant tumor group and nine (14.5%) in the nonmalignant tumor group could not walk with help at discharge.

The average time taken to obtain the assessed motor skills was similar in both groups (Table 6).

## 4. Discussion

The histopathological subtype of tumor—particularly whether it is malignant or nonmalignant—is not only important in terms of survival outcomes and treatment but must also be considered when planning rehabilitation. Compared to other neurological diseases, relatively few studies evaluated rehabilitation in patients with brain tumors, and they rarely considered whether the tumor is malignant or nonmalignant. Therefore, our primary goal was to compare the functional state and motor skills of patients undergoing primary brain tumor surgery for malignant and nonmalignant primary brain tumors. A secondary aim was to assess the prevalence of complications affecting rehabilitation and its course. In our cohort, the proportions of different tumor types were similar to those reported elsewhere [1,2,3], with about two-thirds nonmalignant with a meningioma predominance and just over one-quarter malignant, with glioblastoma the most common.

We found that patients requiring rehabilitation after malignant brain tumor surgery were generally in a worse functional state before surgery than patients with nonmalignant tumors. Postoperative complications were the same regardless of tumor type; likewise, the time taken to achieve motor functionality was similar in both groups. The proportion of patients walking independently at discharge decreased by 30% in both groups compared with at admission. All nonmalignant tumor patients suffered larger decreases in function, but patients with malignant tumors had lower scores at discharge. These worse functional outcomes for patients with malignant tumors did not affect the LoS or rehabilitation course.

The general condition of the patients and the presence of neurological deficits before surgery are important factors determining the immediate post-surgical state, outcomes from rehabilitation, and overall survival [22,44]. In the entire population of 835 patients operated on for brain tumors, limb paralysis or paresis was present in 6.9% before surgery, and new motor deficits were reported in 4.3% [32]. Among patients requiring postoperative rehabilitation, 21.2% of patients in the nonmalignant tumor group had paralysis/paresis, which were about three times more common in the malignant tumor group. This was reflected in their motor capabilities; 4.5% of subjects could not walk independently in the nonmalignant tumor group, while 19.2% of subjects could not walk independently in the malignant tumor group. The malignant tumor group was also characterized by significantly worse KPS and MRS scores before the operation. Some authors have highlighted the usefulness of preoperative KPS and MRS scores in predicting the postoperative condition and possibility of early discharge [45,46,47,48]. Our results confirmed this relationship. Although, after surgery, the values of the scales used decreased less in the malignant tumor group, all scores were worse in this group at discharge. The published data suggest a broader interpretation of these scales than simply ADL, independence, and performance assessment. Brazil et al. found a correlation between BI and KPS scores and also an association between BI scores and prognosis in patients with glioblastoma; patients with functional independence had a median survival of 9 months, those with moderate disability had a median survival of 5 months, and those with severe disability had a median survival of 4 months [49]. Kreisl et al. reported an association between MRS and overall survival, indicating a relationship between the degree of independence and the survival time [50].

A specific feature of our study was the assessment of basic motor skills: passive and active sitting, standing, and independent gait. Attaining each subsequent level increases ADL, independence, and the possibility of functioning in various spheres of life [7,8,51]. The assessment of these abilities before and after the procedure and at discharge allowed us to evaluate the deterioration in function due to surgery and consider the dynamics of postoperative rehabilitation. We found that motor function was similar between groups before and after surgery, as was the time taken to regain motor function.

Independent gait is one of the main goals of rehabilitation in patients with motor deficits, not only because it determines ADL and independence, but also because it is a reliable indicator of overall health and functional status. Dulaney et al. confirmed that gait speed is similar in predictive value to more complex measures of health and may be a survival factor in patients with brain tumors [52]. Gait efficiency is easily and quickly assessed by clinic staff or during routine physical examination [32]. In our study, all GI values (before and after surgery and at discharge) were lower in the MT group, but the percentage of people walking independently decreased in both groups by about 30% compared with the state at admission. This corresponded to an increase in the rate of paralysis and paresis after surgery. New perioperative motor deficits not only delay follow-up treatment but are also a negative prognostic factor for survival [48,53,54,55,56]. In contrast to motor deficits, which were more common in the malignant tumor group, postoperative complications—surgical and medical—were not related to the tumor type. They affected every fourth patient and had a similar degree of severity in both groups.

LoS is often used as a measure of quality of care, and it is an especially important parameter for patients with a terminal diagnosis and expected survival of only a few months, e.g., those with glioblastoma [54,57]. LoS after most neurosurgical procedures has decreased. For example, the median inpatient stay after craniotomy for tumor resection was 6.8–8.8 days in 1996 [58] and has since decreased significantly. Modern surgical methods such as awake craniotomy allow for a very short postoperative inpatient stay. More patients are now discharged on postoperative day 1 or even on the same day as surgery [45,48,59]. In our study, the LoS after surgery in patients who did not require rehabilitation was 5.1 days [32], consistent with other data [48,60]. For patients who required a prolonged inpatient stay due to postoperative rehabilitation, the mean LoS was over 2 weeks and was not dependent on the tumor type. Additionally, comorbidities, ethnicity, and socioeconomic status have often been cited in small studies as possible confounders of LoS. Socioeconomic disadvantage has been consistently linked to higher mortality rates following high-risk procedures, as well as an increased likelihood of non-home discharge following low-risk procedures [61]. For surgical patients, comorbidities add to their operative risk, particularly in emergency procedures. Several studies have revealed differences in adjusted LoS in medical and surgical admissions as a function of ethnic background and socioeconomic status [62,63]. Our research points to excessively long waiting times for some patients to start definitive treatment after surgery. This is especially important for patients with malignant tumors, whose symptoms can develop or progress very quickly. Our findings also suggest that, despite the favorable prognosis, the rehabilitation needs of patients with nonmalignant tumors are similar to those with malignant tumors. This mirrors the similar mental health burden in patients with nonmalignant and malignant tumors [64]; therefore, practitioners must be aware that “nonmalignant” is not equivalent to lesser physical and mental rehabilitation needs in the vulnerable brain tumor population.

Our assessments of functional state, independence, and performance in the period immediately after surgery are useful for determining nursing needs and for organizing care in the perioperative period. Such an assessment also allows us to better determine the rehabilitation starting point, providing an opportunity to assess the effectiveness and progress of postoperative rehabilitation. In turn, the assessment of functional state at discharge is useful for caregivers and social workers organizing further rehabilitation and appropriate facilities at home. Determining the incidence of complications and the duration of stay in a neurosurgery clinic can be helpful for (re)distributing hospital resources and estimating treatment costs. This work answers the question of which functional parameters are important to consider according to the histopathological subtype of tumor.

Our study had some limitations. First, the group sizes were relatively small and very different, reflecting the malignant and nonmalignant workload in this single institution. The presence of unequal samples sizes reduced the statistical power. However, it is worth emphasizing that our cohort size of 92 participants with 18 months of follow-up compare favorably with the published literature. Thakkar et al. [14] recently presented an up-to-date narrative review of the rehabilitation outcomes of patients operated on for brain tumors, comparing the results of prospective studies in only 10–75 patients and retrospective studies in 35 to 412 participants [7,8,9,11,14,15,17,28]. None of the prospective studies included more participants than our study, and only two retrospective studies presented a larger number of cases. It should be noted that very few studies examined the brain tumors themselves, a problem common to studying other aspects of function after brain tumor resections, such as cognitive science [65].

As a second limitation, we did not formally analyze the histopathology of all 835 tumors; hence, we could not determine whether rehabilitation needs are dependent on the tumor type. Third, we did not include the genetic profiles of the tumors, which may be interesting in the context of functional state. Fourth, follow-up was only the time of inpatient stay, and extended (out of hospital) follow-up would be important in the context of some rehabilitation outcomes. Fifth, surgical access, as well as other technical aspects of surgery determining the postoperative course of patients, were omitted, but these data were not available. Sixth, comorbidities, ethnicity, and socioeconomic status were not taken into account, and these factors are known to influence the course of treatment. The impact of tumor location and initial or repeat surgery on the course and results of rehabilitation is discussed elsewhere [32,33].

## 5. Conclusions

Here, we established that patients requiring rehabilitation after malignant brain tumor surgery were more likely to have neurological deficits and have worse performance and independence both before surgery and at discharge than patients with nonmalignant tumors. Despite this, patients with malignant and nonmalignant tumors have similar rehabilitation needs, whereby the diagnosis of “nonmalignant” does not equate to a lesser need for clinical care, and patient expectations should similarly be appropriately managed despite a relatively benign diagnosis. Lastly, the impact of surgery is similar regardless of tumor type. Since patients with nonmalignant lesions actually suffer more from the effects of surgery due to their better, preoperative starting point; these patients might counterintuitively require more rather than less counseling and support.

## Figures and Tables

**Table 1:** Spectrum of neoplasms in patients rehabilitated after brain tumor surgery (*n* = 92).

Type of Tumor	Type of Neoplasm	WHO Grade	*n*	%
	Hemangioblastoma		5	5.4
Benign	Hemangioma cavernosum		2	2.2
WHO grade I	Meningioma	I	21	22.8
	Schwannoma	I	15	16.3
	Other benign tumors		5	5.4
	Astrocytoma	II	3	3.3
Low-grade gliomas	Diffuse astrocytoma	II	7	7.6
WHO grade II	Oligodendroglioma	II	1	1.1
	Ependymoma	II	3	3.3
	Meningioma atypicum	II	3	3.3
	Central neurocystoma	II	1	1.1
Total nonmalignant tumors			66	71.7
	Anaplastic astrocytoma	III	5	5.4
Malignant	Anaplastic oligodendroglioma	III	1	1.1
WHO grade III, IV	Anaplastic ependymoma	III	2	2.2
	Glioblastoma	IV	16	17.4
	Meningioma anaplasticum	III	1	1.1
	Supratentorial primitive neuroectodermal tumor	IV	1	1.1
Total malignant tumors			26	28.3
Total tumors			92	100

**Table 2:** Comparison of the demographic characteristics of the study participants and time parameters of treatment between those with malignant and nonmalignant neoplasms.

	Malignant *n* = 26	Nonmalignant *n* = 66	*p*-Value
	*n* (%)	*n* (%)	
Male *n* (%)	14 (53.8)	31 (47.0)	0.552
Female *n* (%)	12 (46.2)	35 (53.0)	
	Mean ± SD [range]	Mean ± SD [range]	
Age (years)	51.9 ± 16.9 [23–82]	48.1 ± 17.8 [19–83]	0.352
Overall LoS (days)	19.1 ± 9.7 [8–40]	21.3 ± 15.0 [4–92]	0.491
LoS after surgery (days)	15.2 ± 8.5 [6–31]	17.8 ± 14.7 [2–90]	0.399
Days in ICU after surgery	0.1 ± 0.3 [0–1]	1.0 ± 4.1 [0–31]	0.268
Days of rehabilitation	11.7 ± 6.9 [3–26]	13.3 ± 10.6 [1–53]	0.478

Abbreviations: LoS, length of hospital stay; ICU; intensive care unit.

**Table 3:** Postoperative complications (the Landriel Ibañez classification) and motor deficits.

	Malignant		Nonmalignant		Total		*p*-Value
	*n*	%	*n*	%	*n*	%	
Patients with complications	7	26.9	18	27.3	25	27.2	0.973
Grade I	1	3.8	3	4.5	4	4.3	
Grade II	4	15.4	11	16.7	15	16.3	0.944
Grade III	2	7.7	4	6.1	6	6.5	
Surgical	6	23.1	15	22.7	21	22.8	0.884
Medical	1	3.8	3	4.5	4	4.3	
Temporary	4	15.4	13	19.7	17	18.5	0.468
Permanent	3	11.5	5	9.1	8	8.7	
Plegia/paresis							
Before surgery	17	65.4	14	21.2	31	33.7	<0.001
At discharge	22	84.6	38	57.6	60	85.2	0.014

**Table 4:** Activities of daily living, performance, self-reliance, and gait efficiency before surgery, after surgery, and at discharge.

Variable	Time	Malignant	Nonmalignant	Source	F	*p*-Value
		Mean ± SE	Mean ± SE			
BI	Before surgery	80.8 ± 3.97	93.0 ± 2.49	Group	5.05	0.027
	After surgery	35.4 ± 4.91	40.8 ± 3.08	Time	132.32	<0.001
	At discharge	66.9 ± 4.84	77.0 ± 3.04	G × T	0.63	0.535
KPS	Before surgery	75.4 ± 3.20	87.4 ± 2.01	Group	6.01	0.016
	After surgery	48.1 ± 3.49	52.0 ± 2.19	Time	95.74	<0.001
	At discharge	69.6 ± 3.11	74.1 ± 1.95	G × T	1.91	0.151
MRS	Before surgery	1.9 ± 0.22	1.0 ± 014	Group	10.70	0.002
	After surgery	3.7 ± 0.21	3.4 ± 0.13	Time	91.62	<0.001
	At discharge	2.7 ± 0.22	2.1 ± 0.14	G × T	1.81	0.167
GI	Before surgery	8.0 ± 0.41	9.2 ± 0.26	Group	2.92	0.091
	After surgery	4.1 ± 0.53	4.2 ± 0.33	Time	91.96	<0.001
	At discharge	6.8 ± 0.49	7.6 ± 0.31	G × T	1.23	0.294

Abbreviations: BI, Barthel index; KPS, Karnofsky performance status scale; MRS, modified Rankin scale; GI, gait index; SE, standard error; G × T, interaction effect group × time, F, analysis of variance (ANOVA).

**Table 5:** Functional state before surgery, after surgery, and at discharge.

Motor Skills	Malignant	Nonmalignant	*p*-Value
	*n* (%)	*n* (%)	
Before surgery			
Passive sitting	26 (100%)	66 (100%)	1.000
Active sitting	25 (96.2%)	66 (100%)	0.109
Standing	23 (88.5%)	64 (97.0%)	0.105
Independent gait	21 (80.8%)	63 (95.5%)	0.024
Week after surgery			
Passive sitting	24 (92.3%)	60 (90.1%)	0.830
Active sitting	19 (73.1%)	55 (83.3%)	0.264
Standing	15 (58.7%)	41 (62.1%)	0.695
Independent gait	7 (26.9%)	28 (42.4%)	0.168
At discharge			
Passive sitting	26 (100%)	66 (100%)	1.000
Active sitting	24 (92.3%)	63 (95.5%)	0.548
Standing	22 (84.7%)	60 (90.1%)	0.382
Independent gait	13 (50.0%)	44 (66.7%)	0.138

**Table 6:** Average time taken (days) to obtain the evaluated functional capabilities.

After Surgery	Malignant	Nonmalignant	*p*-Value
(Days)	Mean ± SD [Range]	Mean ± SD [Range]	
Passive sitting	3.9 ± 4.5 [1–18]	4.3 ± 8.5 [1–66]	0.820
Active sitting	4.3 ± 5.3 [1–21]	4.0 ± 4.8 [1–32]	0.793
Independent standing	7.5 ± 8.2 [1–27]	7.5 ± 8.0 [1–45]	1.000
Independent gait	8.3 ± 7.4 [1–26]	8.5 ± 8.0 [1–32]	0.912

## Data Availability

The datasets used and/or analyzed during the current study are available from the corresponding author on reasonable request.
